# Gut microbiota-mediated generation of saturated fatty acids elicits inflammation in the liver in murine high-fat diet-induced steatohepatitis

**DOI:** 10.1186/s12876-017-0689-3

**Published:** 2017-11-29

**Authors:** Shoji Yamada, Nobuhiko Kamada, Takeru Amiya, Nobuhiro Nakamoto, Toshiaki Nakaoka, Masaki Kimura, Yoshimasa Saito, Chieko Ejima, Takanori Kanai, Hidetsugu Saito

**Affiliations:** 1 Division of Pharmacotherapeutics, Faculty of Pharmacy, Keio University, 1-5-30 Shiba-Kohen, Minato-ku, Tokyo 105-8512 Japan; 2 Division of Gastroenterology, Department of Internal Medicine, The University of Michigan Medical School, Ann Arbor, MI 48109 USA; 3 Division of Gastroenterology and Hepatology, Department of Internal Medicine, Keio University, Shinjuku-ku, Tokyo 160-8582 Japan; 4 Research Institute, EA Pharma Co. Ltd, Kawasaki, Kanagawa 210-8681 Japan

**Keywords:** Gut microbiota, Non-alcoholic steatohepatitis, Long-chain saturated fatty acids, Fat accumulation, Fat transportation

## Abstract

**Background:**

The gut microbiota plays crucial roles in the development of non-alcoholic steatohepatitis (NASH). However, the precise mechanisms by which alterations of the gut microbiota and its metabolism contributing to the pathogenesis of NASH are not yet fully elucidated.

**Methods:**

Mice were fed with a recently reported new class of high-fat diet (HFD), steatohepatitis-inducing HFD (STHD)-01 for 9 weeks. The composition of the gut microbiota was analyzed by T-RFLP. Luminal metabolome was analyzed using capillary electrophoresis and liquid chromatography time-of-flight mass spectrometry (CE- and LC-TOFMS).

**Results:**

Mice fed the STHD-01 developed NASH-like pathology within a short period. Treatment with antibiotics prevented the development of NASH by STHD-01. The composition of the gut microbiota and its metabolic activities were markedly perturbed in the STHD-01-fed mice, and antibiotic administration normalized these changes. We identified that long-chain saturated fatty acid and n-6 fatty acid metabolic pathways were significantly altered by STHD-01. Of note, the changes in gut lipidome caused by STHD-01 were mediated by gut microbiota, as the depletion of the gut microbiota could reverse the perturbation of these metabolic pathways. A saturated long-chain fatty acid, palmitic acid, which accumulated in the STHD-01 group, activated liver macrophages and promoted TNF-α expression.

**Conclusions:**

Lipid metabolism by the gut microbiota, particularly the saturation of fatty acids, affects fat accumulation in the liver and subsequent liver inflammation in NASH.

**Electronic supplementary material:**

The online version of this article (10.1186/s12876-017-0689-3) contains supplementary material, which is available to authorized users.

## Background

The number of patients with fatty liver disease (FLD) has been steadily increasing in recent years. FLD due to causes other than excessive alcohol intake is termed non-alcoholic FLD (NAFLD) [1]. In patients with NAFLD, dysregulation of adipokines, insulin resistance, and dyslipidemia lead to fat accumulation in the liver [2–4]. Activation of Kupffer cells and hepatic stellate cells and lipid peroxidation elicit liver inflammation, thereby leading to the development of non-alcoholic steatohepatitis (NASH). The numbers of patients with NAFLD and NASH in Japan are more than 10 million and 1 million, respectively. Thus, it is required to understand the precise mechanism of action and develop better therapeutic treatments for NAFLD and NASH.

Since many environmental and genetic factors are associated with the development and progression of NASH, the precise mechanisms by which NASH develops are still not completely understood [5]. Animal models are useful tools for understanding the pathogenesis of human diseases inducing NASH. Feeding of mice with a high-fat diet (HFD) leads to the development of NASH in mice [6]. A recent accumulating evidence has highlighted that the gut microbiota and its metabolites play pivotal roles in the development of NASH [7]. It has been reported that feeding of a HFD induces the alteration of gut microbial communities, referred to as dysbiosis [8, 9]. HFD-induced dysbiosis impairs the integrity of the intestinal epithelium, and thereby eliciting the systemic dissemination of the gut microbiota and/or microbial products, such as lipopolysaccharides (LPS) [10]. The microbial stimuli activate the production of pro-inflammatory cytokines in the liver, and liver inflammation elicits the development of NASH [10]. In addition to the direct stimulation by microbial components, metabolic alterations caused by dysbiosis are also known to cause NASH development. In db/db mice, gut dysbiosis with accompanied enrichment of the genus *Bacteroides* alters fatty acid metabolism [11]. Fatty acids are absorbed in the intestine and transported to the liver through the portal vein for energy utility. Although fatty acid metabolism is believed to be involved in the pathogenesis of NASH, the precise mechanisms by which high-fat diet-induced dysbiosis affects fatty acid metabolism in the gut and the consequent effect of this imbalance on liver inflammation have not been fully elucidated.

A recent study has reported a new class of HFD, known as steatohepatitis-inducing HFD (STHD)-01. The consumption of STHD-01 promotes the development of NASH-like pathology within a short period of time [12]. However, since STHD-01 is a recently developed diet, it remains unclear whether this diet impacts the gut microbiota and its metabolic activities, promoting NASH development, similar to that of the commonly used HFD. Hence, in the present study, we comprehensively analyzed the alteration of the gut microbiota composition and its metabolic activities as well as potential mechanisms associated with the STHD-01-induced development of NASH-like symptoms.

## Methods

### Animals

SPF C57BL/6J mice were fed a conventional CE-2 diet (CLEA Japan Inc., Tokyo, Japan) until 8 weeks of age. The gut microbiota was normalized by exchanging beddings between cages every 2–3 days for 2 weeks. From 8 weeks of age, the mice were fed the STHD-01 (11% kcal/protein, 72% kcal/fat, and 17% kcal/nitrogen-free extracts; EA Pharma Co. Ltd., Kawasaki, Kanagawa) [12] for 9 weeks. The control group was fed the Standard diet (SD) (AIN-93G) (19% kcal/protein, 12% kcal/fat, and 69% kcal/nitrogen-free extract). In the microbiota-depleted group, the mice fed with the STHD-01 diet were treated with an antibiotic (Abx) cocktail (ceftazidime, Sigma-Aldrich, Tokyo, Japan; C3809-5G plus metronidazole; Sigma-Aldrich M3761-25G, both 1 g/L) from 7 weeks until 17 weeks. The mice were killed at week 17 with isoflurane (Mylan Inc., Nagoya, Japan) [13] and peripheral blood, intestinal tissues, and liver samples were harvested. Histological evaluation was performed in a blind manner by a hepatologist and two pathologists from Keio University Hospital as described previously [14].

### Measurement of disease markers

The levels of aspartate aminotransferase (AST) and alanine aminotransferase (ALT) were measured using the Spotchem EZ (Sp-4430, Arkrey USA Inc., MN). The levels of triiodothyronine (T3), thyroxin (T4) and monocyte chemoattractant protein (MCP)-1 were measured with an enzyme-linked immunosorbent assay kit (T3 and T4, Alpha Diagnostic Intl. Inc., Antonio, TX. MCP-1, R&D System, Inc., Minneapolis, MN). For the measurement of triglyceride (TG) in the liver tissue, a Folch solution (2:1 chloroform: methanol; Wako Pure Chemical Industries Ltd., Tokyo, Japan; 4 mL) was added to each liver tissue sample (0.1 g), which was then homogenized. After adding and mixing with 0.5% NaCl (1 mL), the mixture was centrifuged at 180 g at 20 °C for 20 min. The lower layer was obtained and vacuum-dried, and then 1 mL of isopropanol (Wako Pure Chemical Industries Ltd.) was added to the precipitate. The levels of TG were measured using the Pureauto S TG-N (Sekisui Medical Co., Ltd., Tokyo, Japan). At week 15, mice were fasted 1 day before measuring the levels of fasting blood glucose. The levels of fasting blood glucose were measured with GT-1640 (Arkrey USA Inc.). The plasma levels of insulin were measured with mouse insulin enzyme-linked immunosorbent assay kit (Shibayagi Co.,Ltd., Gunma, Japan).

### Microbiome analysis

At week 17, feces were obtained from the mice and resuspended in a phosphate-buffered saline solution (PBS) (0.1 g/mL). The fecal suspension was crushed using the Bug Crashar (Taitec GM-01, Saitama, Japan) at maximal rotation for 10 min. The sample was incubated on ice for 5 min and centrifuged at 2300 g at 4 °C for 1 min. The supernatant (500 μL) was placed in another tube and vortexed with 100 μL of 10% SDS and 500 μL of phenol/chloroform/isoamylalcohol. Then, the sample was centrifuged at 20,000 g at 20 °C for 3 min. The supernatant was treated with chloroform/isoamylalcohol and then isoamylalcohol alone, and the DNA pellet was resuspended in 100 μL Tris/ethylenediamine tetraacetic acid buffer (TE) (Sigma) and 0.5 μL RNase A (Qiagen, Hilden, Germany). The DNA was further purified using the Template Preparation Kit (Roche, Basel, Switzerland).

The obtained DNA was analyzed by a terminal restriction fragment length polymorphism analysis (TechnoSuruga Laboratory Co., Ltd., Shizuoka, Japan). The DNA was amplified with fluorescence-labeled primers, and the amplified DNA was then treated with the restriction enzyme BS/I (Takara Bio Inc., Tokyo, Japan) and analyzed using the ABI Prism 3130xl DNA Sequencer (Applied Biosystems, CA) and Gene Mapper (Applied Biosystems). Cluster analysis was performed using the Gene Maths (Applied Maths, Sint-Martens-Latem, Belgium).

To measure the total number of gut bacteria in the feces, a standard curve using *Escherichia coli* genome DNA (JCM1649T, kindly provided by RIKEN, Saitama, Japan) was drawn and inserted into the pGEM®-T EASY vector system (Promega Co., WI). The sample DNA was amplified with primers 8F (5′ AGAGTTTGATYMTGGCTCAG 3′) and 1510R (5′ TACGGYTACCTTGTTACGACTT 3′). Quantitative polymerase chain reaction (qPCR) was performed using the SYBR Green PCR Master Mix (Applied Biosystems). Quantification was carried out using the CFX96Touch™ (Applied Biosystems). The cycle step was 50 °C × 2 min, 95 °C × 10 min, (95 °C × 30 s, 60 °C × 30 s, 72 °C × 1 min) × 50 cycles.

### Quantitative RT-PCR

RNA extraction from the liver and intestinal tissues was carried out in accordance with a previously described method [15] using Isogen (Wako Pure Chemical Industries, Ltd.). The RNA was transcribed into cDNA using the High Capacity cDNA Reverse Transcription kit (Applied Biosystems), and qPCR was performed using the SYBR Green PCR Master Mix (Applied Biosystems). Quantification was carried out using the CFX96Touch™ (Applied Biosystems). The cycle step was 50 °C × 2 min, 95 °C × 10 min, (95 °C × 30 s, 60 °C × 30 s, 72 °C × 1 min) × 50 cycles. The primers used are summarized in Additional file 1.

### Luminal metabolomic analysis

The metabolomic analysis was conducted using the Dual Scan package of Human Metabolome Technologies Inc. (HMT; Yamagata, Japan) using liquid chromatography time-of-flight mass spectrometry (LC-TOFMS) and capillary electrophoresis (CE)-TOFMS for ionic and non-ionic metabolites, respectively, on the basis of the methods described elsewhere [16, 17]. Briefly, for extracting ionic metabolites, approximately 50 mg of feces sample was dissolved in MilliQ water with ratio of 1:9 (*w*/*v*). After centrifugation, 20 μL of the supernatant was suspended with 20 μL of internal standard solution (HMT, Tsuruoka, Yamagata, Japan) and 80 μL of MilliQ water. The solution was then centrifugally filtered through a Millipore 5000-Da cutoff filter (UltrafreeMC-PLHCC, HMT) to remove macromolecules (9100 × g, 4 °C, 60 min) for subsequent analysis with CE-TOFMS. For extracting non-ionic metabolites, approximately 50 mg of feces sample was dissolved in 1 mL of methanol containing internal standard (HMT). After centrifugation, 300 μL of the supernatant was moved into glass vial for evaporation under nitrogen gas and reconstituted with 300 μL of 50% isopropanol (*v*/v) for subsequent analysis with LC-TOFMS. CE-TOFMS analysis was performed using a CE capillary electrophoresis system with a 6210 time-of-flight mass spectrometer, 1100 isocratic high-performance liquid chromatography (HPLC) pump, G1603A CE-MS adapter kit, and G1607A CE-ESI-MS sprayer kit (Agilent Technologies, Waldbronn, Germany). The G2201AA ChemStation software version B.03.01 for CE (Agilent Technologies) was used to control the systems, which were connected by a fused silica capillary (50 μm i.d. × 80 cm total length) with a commercial electrophoresis buffer (H3301–1001 and H3302–1021 for cation and anion analyses, respectively; HMT) as the electrolyte. The spectrometer was scanned from m/z 50 to 1000. LC-TOFMS analysis was performed using an LC System (Agilent 1200 series RRLC system SL) equipped with a 6230 time-of-flight mass spectrometer (Agilent Technologies). The systems were controlled by the G2201AA ChemStation software version B.03.01 for CE equipped with an octadecylsilyl column (2 × 50 mm, 2 μm). Peaks were extracted using an automatic integration software, MasterHands (Keio University, Tsuruoka, Yamagata, Japan), to obtain peak information, including m/z, peak area, and migration time (MT) for CE-TOFMS and retention time (RT) for LC-TOFMS analyses. The following were excluded: signal peaks corresponding to isotopomers, adduct ions, and other product ions of known metabolites. The HMT metabolite database was used to annotate the remaining peaks on the basis of their m/z values with the MTs and RTs determined by TOFMS. Areas of the annotated peaks were then normalized based on internal standard levels and sample amounts to obtain relative levels of each metabolite. Hierarchical cluster analysis and principal component analysis were conducted using HMT’s proprietary software, PeakStat and SampleStat, respectively. Detected metabolites were plotted on metabolic pathway maps using the VANTED software.

### Liver cell preparation

The livers were perfused through the portal vein with fluorescence-activated cell sorting (FACS) buffer [1 g bovine serum albumin (BSA) in 500 mL PBS] and then minced well. The filtrate was centrifuged at 50 g for 1 min, and the supernatant was washed once. The cells were suspended in 25% Percoll and overlaid in 50% Percoll to distinguish resident macrophages from monocytes. After centrifugation at 2000 rpm for 20 min, the cells were collected from the middle layer and were washed and resuspended in an RPMI1640 medium. Flow cytometry was performed as described previously [18]. Briefly, the cells were collected from the upper phase of a Percoll gradient. After blocking with anti-FcR (CD16/32, BD bioscience, NJ) for 20 min, the cells were incubated with specific monoclonal antibodies at 4 °C for 30 min. The liver mononuclear cells were gated as 7-AAD negative CD45.2-FITC (BD bioscience) positive cells. Liver macrophages were stained with PE-conjugated anti-mouse F4/80 mAb and antigen-presenting cell (APC)-conjugated anti-mouse CD11b mAb (both from BD biosciences). Background fluorescence was assessed by staining with the relevant isotype control Abs. Stained cells were analyzed by flow cytometry (FACS Cant II, Becton Dickinson Co. Franklin Lakes, NJ), and data were analyzed using the FlowJo software (FlowJo, LLC, Ashland, OR).

### Liver CD11b^+^ mononuclear phagocytes ex vivo culture

Liver CD11b^+^ mononuclear phagocytes were separated from all other mononuclear phagocytes using MicroBeads (Miltenyi Biotec K.K., Japan). Then, separated CD11b^+^ cells were cultured in an RPMI1640 containing 0.1% penicillin/streptomycin (Sigma), supplemented with 10% fetal bovine serum (Biowest SAS, Nualle, France) with or without 200 μM palmitic acid (PA; Wako). After incubation for 20 h, mRNA was collected from the CD11b^+^ cells using Isogen.

### Statistical analysis

Results were shown as mean ± standard errors. A one-way ANOVA followed by the Tukey’s post-hoc test was used for comparisons of multi groups. The Mann-Whitney *U*-test was used for comparisons of two groups. All comparisons were two-sided, and a *P*-value < 0.05 was considered significant. All statistical analyses were performed using the SPSS 22 for Windows (SPSS, IBM Japan, Tokyo, Japan).

## Results

### STHD-01 induced steatohepatitis through alteration of the gut microbiota

**Fig. 1:**
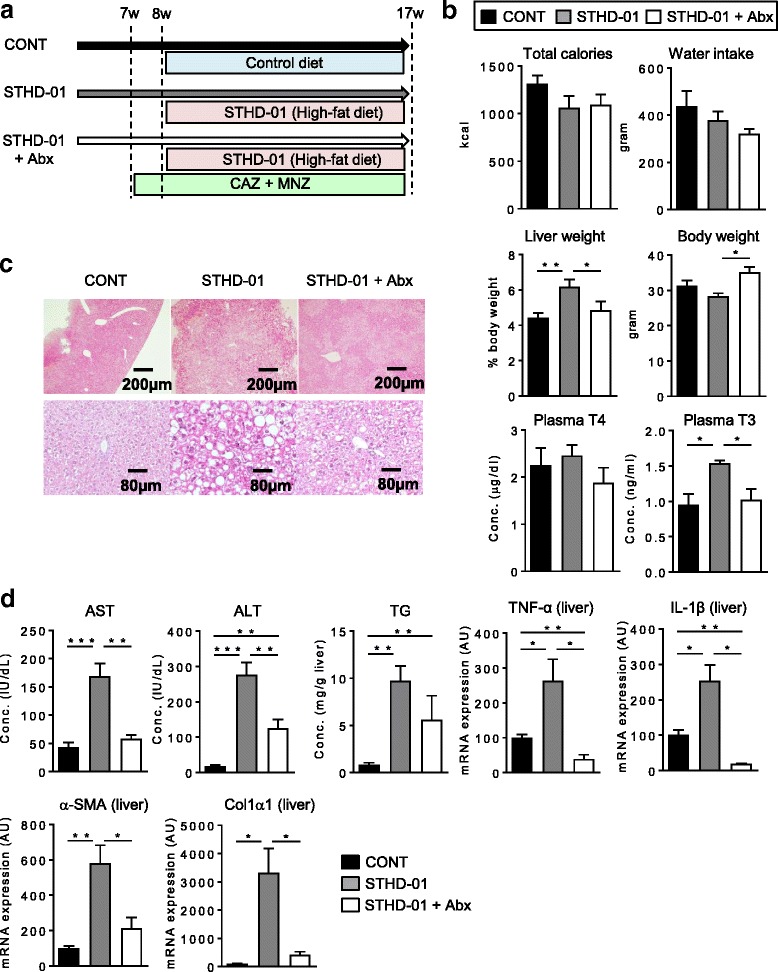
The gut microbiota contributes to the development of NASH induced by STHD-01. **a** Experimental protocol. CAZ; Ceftazidime. MNZ; Metronidazole. **b** Total calorie intake, total water intake, liver/body weight ratios, body weight, and plasma triiodothyronine (T3) and thyroxin (T4) were measured at 17 weeks. **c** Representative histological image of the liver. **d** Plasma aspartate aminotransferase (AST) and alanine aminotransferase (ALT), liver triglyceride (TG), and transcription of TNF-α, IL-1β, α-SMA, and Col1α1 in the liver tissue were measured. **b**, **d** Data are presented as mean ± SEM (*N* = 7). *P < 0.05, ***P < 0.001 by the Tukey’s test. The cut-off value of T3 was 0.16 ng/ml and T4 was 0.5 μg/dl. The cut-off value of AST and ALT were 9 IU/dl. The cut-off value of TG was 0.19 mg/g liver

C57BL/6 J mice were fed a control diet (SD diet; CONT), HFD (STHD-01 diet; STHD-01), or STHD-01 plus Abx (STHD-01 + Abx) for 9 weeks (Fig. 1a). Total calorie intake and total water intake were not different between the three groups (Fig. 1b). The STHD-01 HFD-fed mice exhibited an increased liver mass without an increase in body weight (Fig. 1b). The levels of fasting blood glucose at the week 15 and the plasma levels of insulin were not different between the CONT and HFD group (Additional file 2). Plasma T4, thyroxine, was not altered by STHD-01 (Fig. 1b). In contrast, T3, a thyroid hormone metabolized from T4, was significantly elevated in the STHD-01-fed mice (Fig. 1b). Administration of Abx significantly improved the increased liver weight and plasma T3 induced by STHD-01, suggesting that these changes are mediated by gut dysbiosis induced by STHD-01 (Fig. 1b). Likewise, the STHD-01 group showed significantly larger and more numerous fat droplets throughout the liver (Fig. 1c). Fat droplets were found in both the pericentral and periportal regions (Fig. 1c and Additional file 3). Significant numbers of ballooning and Mallory bodies were observed in the periportal region in the STHD-01 group (Additional file 3). Moreover, several fibrotic extensions were observed in the STHD-01 group (Fig. 1c and Additional file 3). Not only the lipid accumulation but also the STHD-01 induced the infiltration of inflammatory cells in the peripheral region in the liver (Fig. 1c). Notably, these pathological changes in the liver seen in the STHD-01-fed mice were not developed when the mice were administrated with Abx (Fig. 1c). Consistent with these histological changes, the STHD-01-fed mice exhibited increased levels of AST and ALT in the plasma and TG in the liver (Fig. 1d). Moreover, transcription of inflammatory markers tumor nuclear factor (TNF) -α and Interleukin (IL) -1β; and fibrosis markers α-smooth muscle antigen (α-SMA) and α1 type 1 collagen (Col 1α1) mRNAs in the liver were dramatically up-regulated in the STHD-01-fed mice (Fig. 1d). These changes were not observed in mice that receive Abx, suggesting that STHD-01-induced alteration of the gut microbiota plays a critical role in the development of steatohepatitis.

### Metabolic activities of the gut microbiota were dramatically altered in the STHD-01-fed mice

**Fig. 2:**
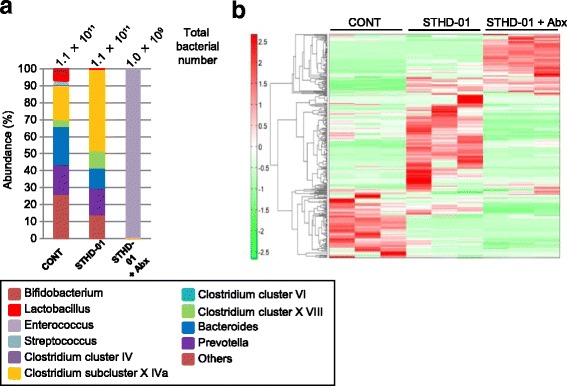
Gut microbiome and luminal metabolomic analyses in NASH mice. **a** Fresh fecal samples were obtained from each group of mice (*N* = 7) on week 17. Microbial. DNA was extracted from feces and analyzed by T-RFLP. The abundance of bacterial genus is indicated. The total number of bacteria (/g feces) is shown at the top of each column. The cut-off value of total number of bacteria was 1.0 × 10^6^ /g feces (**b**) Fresh fecal samples were obtained from each group of mice (3 individual mice) on week 17 and analyzed using capillary electrophoresis time-of-flight mass spectrometry (CE-TOFMS) and liquid chromatography TOFMS (LC-TOFMS). The hierarchical cluster analysis is shown with a heat map of the metabolite

**Fig. 3:**
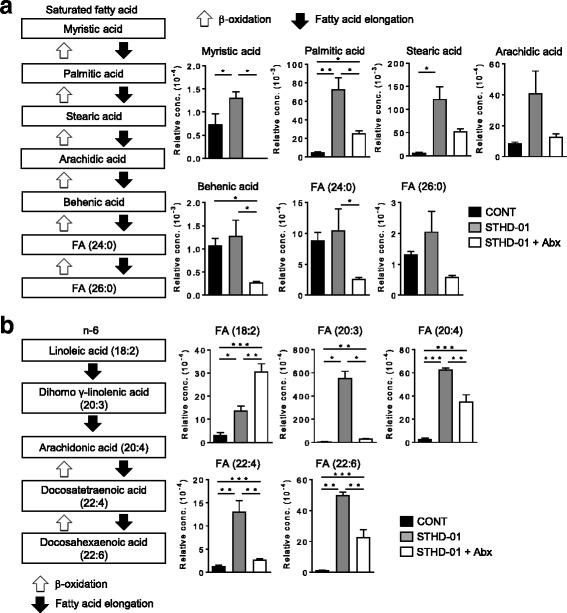
Alteration of fatty acid metabolism induced by STHD-01. Changes in the luminal metabolites categorized into long-chain saturated fatty acids longer than C14 (**a**) and into n-6 unsaturated fatty acids (**b**) are shown. Data are presented as mean ± SEM (*N* = 3). *P < 0.05, **P < 0.01, ***P < 0.001 by the Tukey’s test

Next, we analyzed the composition of the gut microbiota in all three groups. The total number of bacteria in the feces was not affected by feeding of STHD-01 (Fig. 2a). In contrast, treatment with Abx dramatically reduced the number of gut microbes (Fig 2a). After feeding of STHD-01, an abundance of *Bifidobacterium, Enterococcus,* and *Bacteroides* genera decreased, while an abundance of *Clostridium* subclusters XIVa and XVIII increased (Fig. 2a). The treatment of Abx almost completely deleted these major genera of bacteria found in the STHD-01 group, and the genus of *Enterococcus* was dominated within the gut microbiota after the Abx administration. To address the influence of STHD-01-induced dysbiosis on the metabolic activities of the gut microbiota, we analyzed the gut metabolome. Lipid metabolites in the feces were analyzed by liquid LC-TOFMS and hydrophilic metabolites by CE-TOFMS. The detected peaks were categorized into glycolysis/gluconeogenesis, pentose-phosphate, tricarboxylic acid (TCA) cycle, urea cycle, purine-pyrimidine, coenzyme, amino acids, acyl-carnitine, and fatty acid pathways and were included in a pathway map (Additional file 4 A). There were also many detected metabolite peaks that were not categorized into any aforementioned metabolic pathways (Additional file 4 B–C). The relative area ratios of the three experimental groups were compared and shown in a heatmap with hierarchical clustering. This cluster analysis revealed that the luminal metabolic profiles in all three experimental groups were completely different (Fig. 2b). Among the largely different metabolites found in the STHD-01 group, we attempted to identify metabolic pathways in which the metabolites were constantly elevated throughout the pathway. We found that the pathways of long-chain saturated fatty acids, fatty acids longer than C14, and n-6 unsaturated fatty acids were constantly elevated in the STHD-01 group compared with the control group. Strikingly, the elevation of these pathways was normalized to control levels after the treatment with Abx, suggesting that these metabolic pathways were up-regulated due to gut dysbiosis induced by STHD-01 (Figs. 2b and 3).

### Saturated long-chain fatty acids promoted liver inflammation through activation of migratory macrophages in the liver

**Fig. 4:**
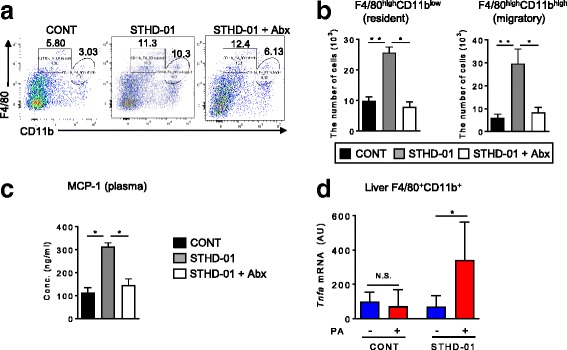
Migratory macrophages in the liver produce TNF-α in response to palmitic acid. At week 17. All three groups of mice were sacrificed, and liver mononuclear cells were isolated. **a** Representative fluorescence-activated cell sorting (FACS) plot for liver macrophages. CD45.2^+^7-AAD^−^ liver mononuclear cells were further expanded by CD11b and F4/80. F4/80^high^CD11b^low^ resident macrophages (square area) and F4/80^high^CD11b^high^ migratory macrophages (circle area) are shown. **b** The absolute number of resident and migratory macrophages in the liver. Data are presented as mean ± SEM (*N* = 7). *P < 0.05 by the Tukey’s test. **c** Monocyte chemoattractant protein (MCP)-1 level in plasma (*N* = 3). (D) Isolated liver CD11b^+^ macrophages were stimulated with palmitic acid (PA) (5.12*10^−3^ mg/mL) for 20 h. The transcription of *Tnfa* mRNA was measured by quantitative polymerase chain reaction (qPCR). The expression of *Tnfa* mRNA was normalized to that of GAPDH. Data are presented as mean ± SEM (*N* = 3). *P < 0.05 by the Mann-Whitney *U*-test

So far, we have found that STHD-01-induced gut dysbiosis altered microbial metabolic activities, and therefore resulted in an accumulation of saturated long-chain fatty acids and n-6 unsaturated fatty acids. However, it remains unclear whether these metabolic changes in the gut lead to liver inflammation. To investigate the detailed phenotype of liver inflammation induced by the STHD-01, we next analyzed the macrophage populations, which play central roles in the development of inflammation in the liver [19]. Macrophages in the liver can be subdivided into resident and migratory macrophages [20]. Although both macrophage subsets express F4/80 and CD11b, resident macrophages reveal F4/80^high^CD11b^low^ phenotypes, and migratory macrophages show F4/80^high^CD11b^high^ phenotypes (Fig. 4a). The number of F4/80^high^CD11b^high^ migratory macrophages was significantly elevated in the STHD-01 group (Fig. 4b). Of note, the migration of F4/80^high^CD11b^high^ macrophages into the liver, which is elicited by STHD-01, was significantly decreased by Abx treatment (Fig. 4b). Next, we examined the plasma level of MCP-1, a chemokine that is responsible for the recruitment of monocytes from the bone marrow to the blood, thereby leading to the migration of monocytes to inflammatory tissue sites. Consistent with the number of migratory macrophages in the liver, plasma MCP-1 level was significantly elevated in the STHD-01 group and it was suppressed by the administration of Abx (Fig. 4c). We next asked the impact of long-chain saturated fatty acids in the induction of liver inflammation. CD11b^+^ macrophages were isolated from the liver of the control (CONT), STHD-01-fed (STHD-01). Isolated CD11b^+^ macrophages were then stimulated with palmitic acids (PA), in which the level was correlated to liver inflammation induced by STHD-01 (Figs. 1c, 2, and 3). We measured the transcription of *Tnfa* mRNA as a marker of liver inflammation. In the CONT group, PA stimulation did not induce *Tnfa* expression in liver macrophages (Fig. 4d). In contrast, PA stimulation significantly induced the expression of *Tnfa* in liver macrophages isolated from STHD-01 (Fig. 4d). Since migratory macrophages were dominated within CD11b^+^ mononuclear cell (MNC)s in the STHD-01 fed mice (Fig. 4a-d), this result indicated that PA could activate migratory, but not the resident, macrophages in the liver. PA was able to induce *Tnfa* expression in liver CD11b^+^ macrophages isolated from this group of mice. This result suggests that STHD-01-induced liver inflammation leads to the increased F4/80^high^CD11b^high^ macrophage migration in the liver. Lipid mediators, which are generated by the gut microbiota, such as PA, promote the progression of liver inflammation by activating the migrated macrophages.

## Discussion

**Fig. 5:**
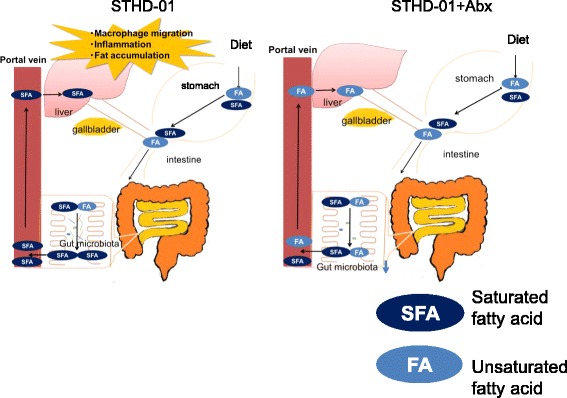
Alternation of the saturated fatty acids by the gut microbiota. Diagram of the current study. Upon the feeding of STHD-01, unsaturated fatty acids (FA) are metabolized to saturated FAs (SFA) by the gut microbiota. SFAs are absorbed from the intestine and accumulate in the liver. The accumulation of SFAs in the liver will promote liver inflammation. On the other hand, the depletion of the gut microbiota by antibiotics results in lower levels of SFA generation and absorption in and from the intestine. Hence, the accumulation of fat and inflammation in the liver are attenuated in the antibiotics-treated mice

The gut microbiota plays a critical role in the pathogenesis of NASH [21]. Here, we comprehensively analyzed the impact of feeding of a new class of HFD, STHD-01, on the gut microbiota and subsequent development of steatohepatitis. As reported previously in conventional HFDs, feeding of STHD-01 induced significant alterations in the gut microbial composition and subsequent luminal metabolic profiles. Depletion of the gut microbiota by treatment of Abx significantly improved the STHD-01-induced mal-metabolic profile in the gut and attenuated liver inflammation. Our present study highlighted a role of the long-chain saturated fatty acid, PA, which accumulated due to STHD-01-induced dysbiosis, in the induction of liver inflammation (Fig. 5).

It has been extensively reported that the intake of HFD causes NASH in experimental animal models [6]. In the present study, we used a recently developed novel steatohepatitis-inducing HFD, STHD-01 [12], to induce NASH. This novel HFD contains a high amount of cholesterol, which is not contained in conventionally used HFDs, and induces the development of severe NASH, while conventionally-used HFDs only induce mild to moderate NASH in a shorter period of time [22]. Another specific feature of STHD-01 is that STHD-01 does not affect fasting blood glucose levels (Additional file 2). While certain type of diet, such as methionine- and choline-deficient diet (MCD), can also cause an advanced NASH [23], this diet decreases fasting blood glucose levels in experimental animals [24]. Since non-overweight human patients with NAFLD do not show decreased fasting blood glucose levels compared to non-fatty liver disease patients [25], STHD-01 is a better approximation of the clinical condition. One obvious difference in the phenotypes between the mice fed with the STHD-01 and the conventional HFD is body weight gain. Contrary to conventional HFD feeding, the STHD-01-fed mice did not gain body weight while experiencing liver weight gain and lipid accumulation (Fig. 1). These phenotypes may in part be explained by the increased plasma T3 levels, which increase energy expenditure in brown adipose tissues. It has been demonstrated that bile acids generated by the metabolism of dietary fat activate the G-protein-coupled receptor (TGR) 5, which in turn stimulates Iodothyronine Deiodinase (DIO) 2 activity. DIO2 metabolizes T4 and converts it into T3 [26]. In our model, the level of total bile acids in the plasma was significantly elevated in the STHD-01-fed mice (data not shown), suggesting that the increased bile acids subsequently increase T3-mediated energy expenditure in brown adipose tissues and therefore prevent body weight gain.

Consistent with previous observations [27–29], STHD-01-induced NASH pathology was mediated by an alteration of the gut microbiota and its metabolic activities. In our study, feeding STHD-01 decreased the occurrence of *Bifidobacterium, Lactobacillus, Enterococcus, Streptococcus* and *Bacteroides*, while increasing the presence of *Clostridium* subclusters XIVa and XVIII. The decline in *Bacteroides* in FLD has been reported previously in ob/ob obese mice [11], and a decrease in *Bacteroides* and an increase in *Streptococcus* were observed in patients with hepatitis compared with healthy subjects [30]. The luminal metabolomic analysis showed significant alterations of the luminal metabolic profiles of the mice when fed with the STHD-01. We identified two major pathways (saturated fatty acid and n-6 fatty acid pathways) as candidates of metabolic pathways overrepresented in the STHD-01 group. Although saturated and unsaturated fatty acids are contained in the diet, the overrepresentation of these lipid metabolism pathways was markedly normalized by the depletion of the gut microbiota. This fact indicates that the accumulation of fatty acids is not merely caused by the diet; rather, the generation of saturated fatty acid is enhanced by the gut microbiota. Consistent with this notion, it has been reported that certain types of gut bacteria can saturate fatty acids [31–33]. However, it is still possible that other bacteria metabolites beside fatty acids are involved in the development of NASH induced by STHD-01. Since the involvement of saturated fatty acids in fatty liver disease has already been reported [34], this was not so unique feature caused by STHD-01. This suggests that different types of HFDs (e.g., different formulations of fatty acids) may bring about a similar microbial/metabolic shift. However, since STHD-01 causes more severe NASH, there might be yet to be determined differences in the abundance of specific microbes and/or metabolites between animals receiving STHD-01 compared to those on conventionally used HFDs. We found that migratory macrophages were significantly increased in the liver upon feeding of the STHD-01. The recruitment of migratory macrophages was significantly suppressed by antibiotics treatment, suggesting that the gut microbiota and their metabolites are indispensable for the macrophage recruitment to the liver. Not only the migration of macrophages, but also the gut microbiota is required for the activation of the migratory macrophages in the liver. Saturated fatty acids, such as PA, which are accumulated upon the feeding of STHD-01 in a gut microbiota dependent manner, can activate migratory macrophages in the liver.

## Conclusions

The gut microbiota and its lipid metabolism play a central role in the pathogenesis of NASH induced by a novel steatohepatitis-inducing STHD-01. Comprehensive experiments that utilize multi-OMICS “dry” analyses (gut microbiome and metabolomic analysis) together with “wet” immunological approaches will allow us to understand the precise mechanisms of development of this disease. Targeting the gut microbiota to modify the metabolism of fatty acids might be a new preventive or therapeutic approach in NAFLD and NASH.

## Additional files

Additional file 1: Primers used in this study. (PDF 32 kb)

Additional file 2: The levels of fasting blood glucose at week 15 and plasma levels of insulin. The levels of fasting blood glucose at week 15 and plasma level of insulin were shown CONT group and STHD-01 group. Data are presented as mean ± SEM (*N* = 4). The cut-off value of fasting blood glucose is 9 mg/dl. The cut-off value of plasma level of insulin is 3.12 ng/ml. (PDF 5 kb)

Additional file 3: Liver histology characteristic of non-alcoholic steatohepatitis (NASH). Upon feeding of steatohepatitis-inducing high-fat diet (STHD-01), ballooning, Mallory-Denk body, fibrosis, inflammatory cell infiltration in the periportal regions and fat accumulation both in the pericentral and periportal regions were observed in the liver. (PDF 72 kb)

Additional file 4: Schematic illustration of the substances detected in the metabolomic analysis of the metabolic pathways. (A) Global metabolomic profiling comparing the detectable molecules in the feces among the 3 experimental groups was performed (*N* = 3 in each group) to determine how different gut bacteria metabolize food. Lipid metabolites in the feces were analyzed using liquid chromatography time-of-flight mass spectrometry (LC-TOFMS), and hydrophilic metabolites were analyzed by capillary electrophoresis time-of-flight mass spectrometry (CE-TOFMS). We identified 225 peaks (158 cations and 67 anions) of hydrophobic metabolites by CE-TOFMS, 115 peaks (65 positives and 50 negatives) of hydrophilic metabolites by LC-TOFMS, and 340 candidate compounds (CE-TOFMS 225 and LC-TOFMS 115). These detected peaks were categorized into glycolysis/glyconeogenesis, pentose-phosphate, tricarboxylic acid (TCA) cycle, urea cycle, purine-pyrimidine, coenzyme, amino acids, acyl-carnitine, and fatty acid pathways and were included in a pathway map. Pathway mapping shows a quantitative comparison of the molecules in the 3 experimental groups. (B) The 38 selected metabolites that were increased specifically in antibiotics treated group compared to the control or STHD-01 groups. (*N* = 3 in each group) Among these metabolites, 6 metabolites were detected in STHD-01 + Abx group in high concentration, while these were undetectable in the STHD-01 group. The concentration of 32 metabolites were as >3-fold higher in STHD-01 + Abx group than that in the STHD-01 group. (C) The 78 selected metabolites that were increased specifically in the STHD-01 group compared to the STHD-01 + Abx group. (*N* = 3 in each group) Among these metabolites, 16 metabolites were detected in the STHD-01 group in high concentration, while these were undetectable in the STHD-01 + Abx group. The concentration of 62 metabolites were as >3-fold higher in the STHD-01 group than that in the STHD-01 + Abx group. (ZIP 552 kb)

## Abbreviations

Abx: Antibiotics
ALT: Alanine aminotransferase
APC: Antigen-presenting cell
AST: Aspartate aminotransferase
BSA: Bovine serum albumin
CE: Capillary electrophoresis
Col 1α1: α1 type 1 collagen
CONT: Control diet
DIO: Iodothyronine deiodinase
FACS: Fluorescence-activated cell sorting
FLD: Fatty liver disease
HFD: High-fat diet
HPLC: High-performance liquid chromatography
IL: Interleukin
LC-TOFMS: Liquid chromatography time-of-flight mass spectrometry
LPS: Lipopolysaccharides
MCD: Methionine- and choline-deficient diet
MCP-1: Monocyte chemoattractant protein
MNC: Mononuclear cell
MT: Migration time
NAFLD: Non-alcoholic FLD
NASH: Non-alcoholic steatohepatitis
PA: Palmitic acid
PBS: Phosphate-buffered saline solution
qPCR: Quantitative polymerase chain reaction
RT: Retention time
SD: Standard diet
SFA: Saturated long-chain fatty acid
STHD: Steatohepatitis-inducing HFD
T3: Triiodothyronine
T4: Thyroxin
TCA: Tricarboxylic acid
TE: Tris/ethylenediamine tetraacetic acid buffer
TG: Triglyceride
TGR: The G-protein-coupled receptor
TNF: Tumor nuclear factor
α-SMA: α-smooth muscle antigen

## References

[1] Bellentani S, Sacoccio G, Masutti F, Crocè LS, Brandi G, Sasso F, Cristanini G, Tiribelli C (2000). Prevalence of and risk factors for hepatic steatosis in northern Italy. Ann Intern Med.

[2] Arata M, Nakajima J, Nishimata S, Nagata T, Kawashima H (2014). Nonalcoholic steatohepatitis and insulin resistance in children. World J Diabetes.

[3] Moraes Ados S, Pisani L, Corgosinho FC, Carvalho LO, Masquio DC, Jamar G, Sanches RB, Oyama LM, Dâmaso AR, Belote C (2013). The role of leptinemia state as a mediator of inflammation in obese adults. Horm Metab Res.

[4] Imajo K, Hyogo H, Yoneda M, Honda Y, Kessoku T, Tomeno W, Ogawa Y, Taguri M, Mawatari H, Nozaki Y (2014). LDL-migration index (LDL-MI), an indicator of small dense low-density lipoprotein (sdLDL), is higher in non-alcoholic steatohepatitis than in non-alcoholic fatty liver: a multicenter cross-sectional study. PLoS One.

[5] Tilg H, Moschen AR (2010). Evolution of inflammation in nonalcoholic fatty liver disease: the multiple parallel hits hypothesis. Hepatology.

[6] Ito M, Suzuki J, Tsujioka S, Sasaki M, Gomori A, Shirakura T, Hirose H, Ito M, Ishihara A, Iwaasa H (2007). Longitudinal analysis of murine steatohepatitis model induced by chronic exposure to high-fat diet. Hepatol Res.

[7] Yoshimoto S, Loo TM, Atarashi K, Kanda H, Sato S, Oyadomari S, Iwakura Y, Oshima K, Morita H, Hattori M (2013). Obesity-induced gut microbial metabolite promotes liver cancer through senescence secretome. Nature.

[8] De Minicis S, Rychlicki C, Agostinelli L, Saccomanno S, Candelaresi C, Trozzi L, Mingarelli E, Facinelli B, Magi G, Palmieri C, Marzioni M (2014). Dysbiosis contributes to fibrogenesis in the course of chronic liver injury in mice. Hepatology.

[9] Hildebrandt MA, Hoffmann C, Sherrill-Mix SA, Keilbaugh SA, Hamady M, Chen YY, Ahima RS, Bushman F, Wu GD (2009). High-fat diet determines the composition of the murine gut microbiome independently of obesity. Gastroenterology.

[10] Imajo K, Fujita K, Yoneda M, Nozaki Y, Ogawa Y, Shinohara Y, Kato S, Mawatari H, Shibata W, Kitani H (2012). Hyperresponsivity to low-dose endotoxin during progression to nonalcoholic steatohepatitis is regulated by leptin-mediated signaling. Cell Metab.

[11] Ridaura VK, Faith JJ, Rey FE, Cheng J, Duncan AE, Kau AL, Griffin NW, Lombard V, Henrissat B, Bain JR (2013). Gut microbiota from twins discordant for obesity modulate metabolism in mice. Science.

[12] Ejima C, Kuroda H, Ishizaki S (2016). A novel diet-induced murine model of steatohepatitis with fibrosis for screening and evaluation of drug candidates for nonalcoholic steatohepatitis. Physiological Reports.

[13] Roustan A, Perrin J, Berthelot-Ricou A, Lopez E, Botta A, Courbiere B (2012). Evaluating methods of mouse euthanasia on the oocyte quality: cervical dislocation versus isoflurane inhalation. Lab Anim.

[14] Takaki Y, Saito Y, Takasugi A, Toshimitsu K, Yamada S, Muramatsu T, Kimura M, Sugiyama K, Suzuki H, Arai E (2014). Silencing of microRNA-122 is an early event during hepatocarcinogenesis from non-alcoholic steatohepatitis. Cancer Sci.

[15] Hibino S, Saito Y, Muramatsu T, Otani A, Kasai Y, Kimura M, Saito H (2014). Inhibitors of enhancer of zeste homolog 2 (EZH2) activate tumor-suppressor microRNAs in human cancer cells. Oncogene.

[16] Ooga T, Sato H, Nagashima A, Sasaki K, Tomita M, Soga T, Ohashi Y (2011). Metabolomic anatomy of an animal model revealing homeostatic imbalances in dyslipidaemia. Mol BioSyst.

[17] Sugiyama K, Ebinuma H, Nakamoto N, Sakasegawa N, Murakami Y, Chu PS, Usui S, Ishibashi Y, Wakayama Y, Taniki N (2014). Prominent steatosis with hypermetabolism of the cell line permissive for years of infection with hepatitis C virus. PLoS One.

[18] Chu PS, Nakamoto N, Ebinuma H, Usui S, Saeki K, Matsumoto A, Mikami Y, Sugiyama K, Tomita K, Kanai T (2013). C-C motif chemokine receptor 9 positive macrophages activate hepatic stellate cells and promote liver fibrosis in mice. Hepatology.

[19] Nakamoto N, Ebinuma H, Kanai T, Chu PS, Ono Y, Mikami Y, Ojiro K, Lipp M, Love PE, Saito H, Hibi T (2012). CCR9+ macrophages are required for acute liver inflammation in mouse models of hepatitis. Gastroenterology.

[20] Hettinger J, Richards DM, Hansson J, Barra MM, Joschko AC, Krijgsveld J, Feuerer M (2013). Origin of monocytes and macrophages in a committed progenitor. Nat Immunol.

[21] Malaguarnera M, Vacante M, Antic T, Giordano M, Chisari G, Acquaviva R, Mastrojeni S, Malaguarnera G, Mistretta A, Li Volti G (2012). Bifidobacterium longum with fructo-oligosaccharides in patients with non alcoholic steatohepatitis. Dig Dis Sci.

[22] Itoh M, Suganami T, Nakagawa N, Tanaka M, Yamamoto Y, Kamei Y, Terai S, Sakaida I, Ogawa Y (2011). Melanocortin 4 receptor-deficient mice as a novel mouse model of nonalcoholic steatohepatitis. Am J Pathol.

[23] Machado MV, Michelotti G, Xie G, Almeida PT, Boursier J, Bohnic B, Guy CD, Diehl AM (2015). Mouse models of diet-induced nonalcoholic steatohepatitis reproduce the heterogeneity of the human disease. PLoS One.

[24] Rinella ME, Green RM (2004). The methionine-choline deficient dietary model of steatohepatitis does not exhibit insulin resistance. J Hepatol.

[25] Omagari K, Kadokawa Y, Masuda J, Egawa I, Sawa T, Hazama H, Ohba K, Isomoto H, Mizuta Y, Hayashida K (2002). Fatty liver in non-alcoholic non-overweight Japanese adults: incidence and clinical characteristics. J Gastroenterol Hepatol.

[26] Watanabe M, Morikawa K, Houten SM, Kaneko-Iwasaki N, Sugizaki T, Horai T, Mataki C, Sato H, Murahashi K, Arita E (2012). Bile acid binding resin improves metabolic control through the induction of energy expenditure. PLoS One.

[27] Jiang C, Xie C, Li F, Zhang L, Nichols RG, Krausz KW, Cai J, Qi Y, Fang ZZ, Takahashi S (2015). Intestinal farnesoid X receptor signaling promotes nonalcoholic fatty liver disease. J Clin Invest.

[28] Vanderhoof JA, Tuma DJ, Antonson DL, Sorrell MF (1982). Effect of antibiotics in the prevention of jejunoileal bypass-induced liver dysfunction. Digestion.

[29] Bergheim I, Weber S, Vos M, Krämer S, Volynets V, Kaserouni S, McClain CJ, Bischoff SC (2008). Antibiotics protect against fructose-induced hepatic lipid accumulation in mice: role of endotoxin. J Hepatol.

[30] Qin N, Yang F, Li A, Prifti E, Chen Y, Shao L, Guo J, Le Chatelier E, Yao J, Wu L (2014). Alterations of the human gut microbiome in liver cirrhosis. Nature.

[31] Takeuchi M, Kishino S, Hirata A, Park SB, Kitamura N, Ogawa J (2015). Characterization of the linoleic acid Δ9 hydratase catalyzing the first step of polyunsaturated fatty acid saturation metabolism in lactobacillus plantarum AKU 1009a. J Biosci Bioeng.

[32] Kishino S, Takeuchi M, Park SB, Hirata A, Kitamura N, Kunisawa J, Kiyono H, Iwamoto R, Isobe Y, Arita M (2013). Polyunsaturated fatty acid saturation by gut lactic acid bacteria affecting host lipid composition. Proc Natl Acad Sci U S A.

[33] Hirata A, Kishino S, Park SB, Takeuchi M, Kitamura N, Ogawa J (2015). A novel unsaturated fatty acid hydratase toward C16 to C22 fatty acids from lactobacillus acidophilus. J Lipid Res.

[34] Reichardt F, Chassaing B, Nezami BG, Li G, Tabatabavakili S, Mwangi S, Uppal K, Liang B, Vijay-Kumar M, Jones D (2017). Western diet induces colonic nitrergic myenteric neuropathy and dysmotility in mice via saturated fatty acid- and lipopolysaccharide-induced TLR4 signalling. J Physiol.

