# Supplementary material for: Gut microbiota-mediated generation of saturated fatty acids elicits inflammation in the liver in murine high-fat diet-induced steatohepatitis
Source: BMC Gastroenterol. 2017 Nov 29;17:136. doi: 10.1186/s12876-017-0689-3 (PMC5708095; doi:10.1186/s12876-017-0689-3)
Supplement: Supplementary file 1 — Primers used in this study. (PDF 32 kb) [file 12876_2017_689_MOESM1_ESM.pdf]

α-SMA	F	GACGCTGAAGTATCCGATAGAACACG
	R	CACCATCTCCAGAGTCCAGCACAAT
Col 1α1	F	GGAGGGCGAGTGCTGTGCTTT
	R	GGGACCAGGAGGACCAGGAAGT
IL-1β	F	TCACAGCAGCACATCAACAA
	R	TGTCCTCATCCTGGAAGGTC
TNF-α	F	CACGCTCTTCTGTCTACTGAACTTC
	R	ATGATCTGAGTGTGAGGGTCTGG
GAPDH	F	GTGTCCGTCGTGGATCTGA
	R	CCTGCTTCACCACCTTCTTGA

Supplemental information 1
