# Supplementary figures and images for: Gut microbiota-mediated generation of saturated fatty acids elicits inflammation in the liver in murine high-fat diet-induced steatohepatitis
Source: BMC Gastroenterol. 2017 Nov 29;17:136. doi: 10.1186/s12876-017-0689-3 (PMC5708095; doi:10.1186/s12876-017-0689-3)

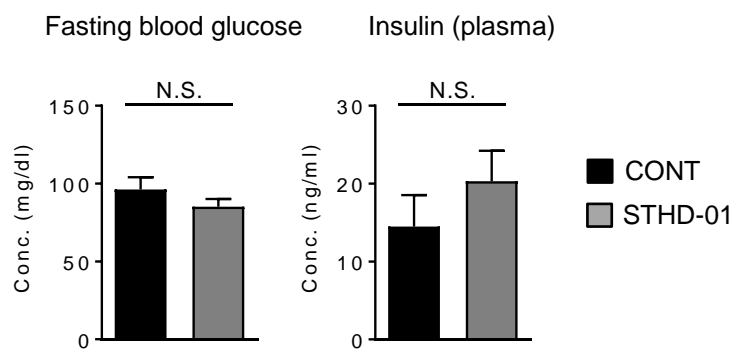

Supplemental information 2

Supplement: Supplementary file 2 — The levels of fasting blood glucose at week 15 and plasma levels of insulin. The levels of fasting blood glucose at week 15 and plasma level of insulin were shown CONT group and STHD-01 group. Data are presented as mean ± SEM (N = 4). The cut-off value of fasting blood glucose is 9 mg/dl. The cut-off value of plasma level of insulin is 3.12 ng/ml. (PDF 5 kb) [file 12876_2017_689_MOESM2_ESM.pdf]

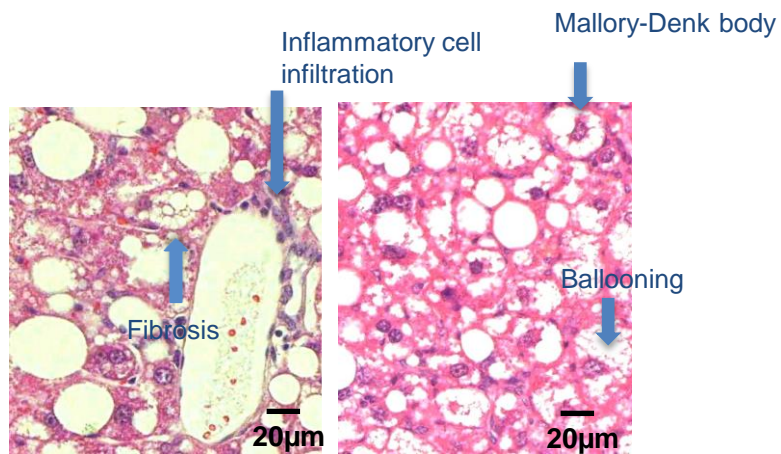

Supplemental information 3

Supplement: Supplementary file 3 — Liver histology characteristic of non-alcoholic steatohepatitis (NASH). Upon feeding of steatohepatitis-inducing high-fat diet (STHD-01), ballooning, Mallory-Denk body, fibrosis, inflammatory cell infiltration in the periportal regions and fat accumulation both in the pericentral and periportal regions were observed in the liver. (PDF 72 kb) [file 12876_2017_689_MOESM3_ESM.pdf]

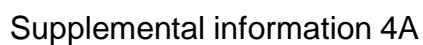

Supplement: Supplementary file 4 — Schematic illustration of the substances detected in the metabolomic analysis of the metabolic pathways. (A) Global metabolomic profiling comparing the detectable molecules in the feces among the 3 experimental groups was performed (N = 3 in each group) to determine how different gut bacteria metabolize food. Lipid metabolites in the feces were analyzed using liquid chromatography time-of-flight mass spectrometry (LC-TOFMS), and hydrophilic metabolites were analyzed by capillary electrophoresis time-of-flight mass spectrometry (CE-TOFMS). We identified 225 peaks (158 cations and 67 anions) of hydrophobic metabolites by CE-TOFMS, 115 peaks (65 positives and 50 negatives) of hydrophilic metabolites by LC-TOFMS, and 340 candidate compounds (CE-TOFMS 225 and LC-TOFMS 115). These detected peaks were categorized into glycolysis/glyconeogenesis, pentose-phosphate, tricarboxylic acid (TCA) cycle, urea cycle, purine-pyrimidine, coenzyme, amino acids, acyl-carnitine, and fatty acid pathways and were included in a pathway map. Pathway mapping shows a quantitative comparison of the molecules in the 3 experimental groups. (B) The 38 selected metabolites that were increased specifically in antibiotics treated group compared to the control or STHD-01 groups. (N = 3 in each group) Among these metabolites, 6 metabolites were detected in STHD-01 + Abx group in high concentration, while these were undetectable in the STHD-01 group. The concentration of 32 metabolites were as >3-fold higher in STHD-01 + Abx group than that in the STHD-01 group. (C) The 78 selected metabolites that were increased specifically in the STHD-01 group compared to the STHD-01 + Abx group. (N = 3 in each group) Among these metabolites, 16 metabolites were detected in the STHD-01 group in high concentration, while these were undetectable in the STHD-01 + Abx group. The concentration of 62 metabolites were as >3-fold higher in the STHD-01 group than that in the STHD-01 + Abx group. (ZIP 552 kb) [file 12876_2017_689_MOESM4_ESM.zip › 12876_2017_689_MOESM4_ESM/Yamada Supplimental Information 4AR3.pdf]
