# Supplementary material for: Gut microbiota-mediated generation of saturated fatty acids elicits inflammation in the liver in murine high-fat diet-induced steatohepatitis
Source: BMC Gastroenterol. 2017 Nov 29;17:136. doi: 10.1186/s12876-017-0689-3 (PMC5708095; doi:10.1186/s12876-017-0689-3)
Supplement: Supplementary file 4 — Schematic illustration of the substances detected in the metabolomic analysis of the metabolic pathways. (A) Global metabolomic profiling comparing the detectable molecules in the feces among the 3 experimental groups was performed (N = 3 in each group) to determine how different gut bacteria metabolize food. Lipid metabolites in the feces were analyzed using liquid chromatography time-of-flight mass spectrometry (LC-TOFMS), and hydrophilic metabolites were analyzed by capillary electrophoresis time-of-flight mass spectrometry (CE-TOFMS). We identified 225 peaks (158 cations and 67 anions) of hydrophobic metabolites by CE-TOFMS, 115 peaks (65 positives and 50 negatives) of hydrophilic metabolites by LC-TOFMS, and 340 candidate compounds (CE-TOFMS 225 and LC-TOFMS 115). These detected peaks were categorized into glycolysis/glyconeogenesis, pentose-phosphate, tricarboxylic acid (TCA) cycle, urea cycle, purine-pyrimidine, coenzyme, amino acids, acyl-carnitine, and fatty acid pathways and were included in a pathway map. Pathway mapping shows a quantitative comparison of the molecules in the 3 experimental groups. (B) The 38 selected metabolites that were increased specifically in antibiotics treated group compared to the control or STHD-01 groups. (N = 3 in each group) Among these metabolites, 6 metabolites were detected in STHD-01 + Abx group in high concentration, while these were undetectable in the STHD-01 group. The concentration of 32 metabolites were as >3-fold higher in STHD-01 + Abx group than that in the STHD-01 group. (C) The 78 selected metabolites that were increased specifically in the STHD-01 group compared to the STHD-01 + Abx group. (N = 3 in each group) Among these metabolites, 16 metabolites were detected in the STHD-01 group in high concentration, while these were undetectable in the STHD-01 + Abx group. The concentration of 62 metabolites were as >3-fold higher in the STHD-01 group than that in the STHD-01 + Abx group. (ZIP 552 kb) [file 12876_2017_689_MOESM4_ESM.zip › 12876_2017_689_MOESM4_ESM/Yamada Supplimental Information 4BR3.pdf]

Categoly		Substance name	Fold vs STHD-01
Amino acid	STHD-01 not detected and STHD-01+Abx 2-folds > CONT	Asn	
		Cys	
	STHD-01+Abx: 3-folds > STHD-01	Gln	3.51
		Carnitine	3.80
		Thr	3.83
		Cystine	4.14
		Argininosuccinic acid	4.76
		Formiminoglutamic acid	6.29
		N-Acetylaspartic acid	7.09
		Creatine	8.81
		Betaine aldehyde_+H_2O	9.05
		Ornithine	29.40
		S-Sulfocysteine	42.95
Hormone	STHD-01 not detected and STHD-01+Abx 2-folds > CONT	Hydroxyprogesterone caproate	
	STHD-01+Abx: 3-folds > STHD-01	Tauroursodeoxycholic acid	81.37
		Taurocholic acid	1068.14
Fatty acid	STHD-01 not detected and STHD-01+Abx 2-folds > CONT	Linolenic acid	
Steroid	STHD-01 not detected and STHD-01+Abx 2-folds > CONT	Mevalonic acid	
Nucleic acid	STHD-01 not detected and STHD-01+Abx 2-folds > CONT	2'-Deoxycytidine	
	STHD-01+Abx: 3-folds > STHD-01	2'-Deoxyguanosine	3.07
Sugar	STHD-01+Abx: 3-folds > STHD-01	Glucuronic acid	33.67
Carnitine	STHD-01+Abx: 3-folds > STHD-01	Acylcarnitine (14:0)	3.37
		Acylcarnitine (16:1)	4.72
		Acylcarnitine (18:2)	6.39
		Acylcarnitine(12:0)	33.48
		Acylcarnitine(14:1)	37.53
		Acylcarnitine(16:2)	64.36
Membrane	STHD-01+Abx: 3-folds > STHD-01	1-Stearoyl-glycero-3-phosphocholine	3.45
		1-Palmitoyl-glycero-3-phosphocholine	3.55
		1-Myristoyl-glycero-3-phosphocholine	4.39
		Phytosphingosine	5.94
Not	STHD-01+Abx: 3-folds > STHD-01	Betaine	3.84
		Ricinoleic acid	3.98
		1-Hexadecyl-2-acetyl-glycero-3-phosphocholine	6.22
		Glucaric acid	8.29
		N^5-Ethylglutamine	9.96
		2-Phenylethylamine	18.28
		Tyramine	77.67

Supplemental information 4B
