# Supplementary material for: Gut microbiota-mediated generation of saturated fatty acids elicits inflammation in the liver in murine high-fat diet-induced steatohepatitis
Source: BMC Gastroenterol. 2017 Nov 29;17:136. doi: 10.1186/s12876-017-0689-3 (PMC5708095; doi:10.1186/s12876-017-0689-3)
Supplement: Supplementary file 4 — Schematic illustration of the substances detected in the metabolomic analysis of the metabolic pathways. (A) Global metabolomic profiling comparing the detectable molecules in the feces among the 3 experimental groups was performed (N = 3 in each group) to determine how different gut bacteria metabolize food. Lipid metabolites in the feces were analyzed using liquid chromatography time-of-flight mass spectrometry (LC-TOFMS), and hydrophilic metabolites were analyzed by capillary electrophoresis time-of-flight mass spectrometry (CE-TOFMS). We identified 225 peaks (158 cations and 67 anions) of hydrophobic metabolites by CE-TOFMS, 115 peaks (65 positives and 50 negatives) of hydrophilic metabolites by LC-TOFMS, and 340 candidate compounds (CE-TOFMS 225 and LC-TOFMS 115). These detected peaks were categorized into glycolysis/glyconeogenesis, pentose-phosphate, tricarboxylic acid (TCA) cycle, urea cycle, purine-pyrimidine, coenzyme, amino acids, acyl-carnitine, and fatty acid pathways and were included in a pathway map. Pathway mapping shows a quantitative comparison of the molecules in the 3 experimental groups. (B) The 38 selected metabolites that were increased specifically in antibiotics treated group compared to the control or STHD-01 groups. (N = 3 in each group) Among these metabolites, 6 metabolites were detected in STHD-01 + Abx group in high concentration, while these were undetectable in the STHD-01 group. The concentration of 32 metabolites were as >3-fold higher in STHD-01 + Abx group than that in the STHD-01 group. (C) The 78 selected metabolites that were increased specifically in the STHD-01 group compared to the STHD-01 + Abx group. (N = 3 in each group) Among these metabolites, 16 metabolites were detected in the STHD-01 group in high concentration, while these were undetectable in the STHD-01 + Abx group. The concentration of 62 metabolites were as >3-fold higher in the STHD-01 group than that in the STHD-01 + Abx group. (ZIP 552 kb) [file 12876_2017_689_MOESM4_ESM.zip › 12876_2017_689_MOESM4_ESM/Yamada Supplimental Information 4C.pdf]

Categoly		Substance name	Fold vs STHD-01+Abx
Amino acid	STHD-01+Abx not detected and STHD-01 2-folds > CONT	Sarcosine	
		N-Acetylglucosamine 6-phosphate	
		3-Aminoisobutyric acid	
	STHD-01: 3-folds > STHD-01+Abx	*N*-Acetylglutamic acid	3.48
		Ala	3.69
		Tyr	6.38
		3-Methylhistidine	7.65
		1-Methyl-4-imidazoleacetic acid	10.37
		Putrescine	14.84
		N-Acetylglucosamine	15.08
		Guanidoacetic acid	21.06
		Glucosamine	23.48
		Succinic acid	28.08
		5-Aminovaleric acid	30.67
		GABA	75.69
hormone	STHD-01+Abx not detected and STHD-01 2-folds > CONT	Taurodeoxycholic acid	
Fatty acid	STHD-01+Abx not detected and STHD-01 2-folds > CONT	Fatty acid (22:5)	
	STHD-01: 3-folds > STHD-01+Abx	Fatty acid (26:0)	3.45
		Fatty acid (24:0)	3.97
		Behenic acid	4.72
		Fatty acid (22:4)	4.86
		Fatty acid (20:3)	16.82
Nucleic acid	STHD-01+Abx not detected and STHD-01 2-folds > CONT	Ribose 5-phosphate	
	STHD-01: 3-folds > STHD-01+Abx	Hypoxanthine	3.05
		Uracil	4.65
		Adenine	7.22
		AMP	10.15
		CMP	10.55
		UMP	20.84
		GMP	26.14
Membrane	STHD-01: 3-folds > STHD-01+Abx	Sphingomyelin (d18:1/18:0)	3.70
		Glycerol 3-phosphate	78.01
Glycerophospholipid	STHD-01: 3 -folds > STHD-01+Abx	Ethanolamine	9.08
Pyruvate	STHD-01: 3-folds > STHD-01+Abx	Malic acid	14.28
Cell signalling	STHD-01+Abx not detected and STHD-01 2-folds > CONT	Lithocholic acid	
		Deoxycholic acid	
		Isovaleric acid	
	STHD-01: 3-folds > STHD-01+Abx	Arachidic acid	3.17
		Heneicosanoic acid	3.20
		Nervonic acid	3.99
		cis-11-Eicosenoic acid	4.82
		Glycocholic acid	4.83
		Cholic acid	5.19
		6-Aminohexanoic acid	6.13
		Cholesterol	6.47
		Ursodeoxycholic acid	10.91
		γ-Butyrobetaine	27.52
		Valeric acid	35.49
		Butyric acid	65.26
Vitamin	STHD-01: 3-folds > STHD-01+Abx	Nicotinic acid	8.01
		Pantothenic acid	8.53
Not	STHD-01+Abx not detected and STHD-01 2-folds > CONT	*N*-Acetylleucine	
		Propionic acid	
		Alloisoleucine	
		Ribulose 5-phosphate	
		N^2-Acetylaminoadipic acid	
		Isopropanolamine	
		2-Hydroxyglutaric acid	
	STHD-01: 3-folds > STHD-01+Abx	Creatinine	3.60
		N-Glycolylneuraminic acid	3.69
		Homovanillic acid	3.77
		4-Pyridoxic acid	4.26
		N-Acetylmuramic acid	4.93
		Glyceric acid	5.10
		N-Acetylneuraminic acid	5.26
		Tartaric acid	5.71
		Oleoyl ethanolamide	6.35
		Sulfotyrosine	6.73
		XC0001	14.93
		Cadaverine	15.52
		p-Hydroxyphenylacetic acid	16.15
		3'-CMP<br>2'-CMP	27.56
		N-Acetylputrescine	27.92
		Isoglutamic acid	30.62
		3-Indoxylsulfuric acid	39.57
		1*H*-Imidazole-4-propionic acid	48.48
		Trimethylamine	58.14
		Phenaceturic acid	71.60

Supplemental information 4C
